# Progesterone Inhibits Epithelial-to-Mesenchymal Transition in Endometrial Cancer

**DOI:** 10.1371/journal.pone.0030840

**Published:** 2012-01-25

**Authors:** Paul H. van der Horst, Yongyi Wang, Ingrid Vandenput, Liesbeth C. Kühne, Patricia C. Ewing, Wilfred F. J. van IJcken, Marten van der Zee, Frederic Amant, Curt W. Burger, Leen J. Blok

**Affiliations:** 1 Department of Obstetrics and Gynaecology, Erasmus University Medical Center Rotterdam, Rotterdam, The Netherlands; 2 Division Gynecologic Oncology, University Hospital Gasthuisberg, Catholic University Leuven, Leuven, Belgium; 3 Department of Pathology, Erasmus University Medical Center Rotterdam, Rotterdam, The Netherlands; 4 Department of Biomics, Erasmus University Medical Center Rotterdam, Rotterdam, The Netherlands; Florida International University, United States of America

## Abstract

**Background:**

Every year approximately 74,000 women die of endometrial cancer, mainly due to recurrent or metastatic disease. The presence of tumor infiltrating lymphocytes (TILs) as well as progesterone receptor (PR) positivity has been correlated with improved prognosis. This study describes two mechanisms by which progesterone inhibits metastatic spread of endometrial cancer: by stimulating T-cell infiltration and by inhibiting epithelial-to-mesenchymal cell transition (EMT).

**Methodology and Principal Findings:**

Paraffin sections from patients with (n = 9) or without (n = 9) progressive endometrial cancer (recurrent or metastatic disease) were assessed for the presence of CD4+ (helper), CD8+ (cytotoxic) and Foxp3+ (regulatory) T-lymphocytes and PR expression. Progressive disease was observed to be associated with significant loss of TILs and loss of PR expression. Frozen tumor samples, used for genome-wide expression analysis, showed significant regulation of pathways involved in immunesurveillance, EMT and metastasis. For a number of genes, such as CXCL14, DKK1, DKK4, PEG10 and WIF1, quantitive RT-PCR was performed to verify up- or downregulation in progressive disease. To corroborate the role of progesterone in regulating invasion, Ishikawa(IK) endometrial cancer cell lines stably transfected with PRA (IKPRA), PRB(IKPRB) and PRA+PRB (IKPRAB) were cultured in presence/absence of progesterone (MPA) and used for genome-wide expression analysis, Boyden- and wound healing migration assays, and IHC for known EMT markers. IKPRB and IKPRAB cell lines showed MPA induced inhibition of migration and loss of the mesenchymal marker vimentin at the invasive front of the wound healing assay. Furthermore, pathway analysis of significantly MPA regulated genes showed significant down regulation of important pathways involved in EMT, immunesuppression and metastasis: such as IL6-, TGF-β and Wnt/β-catenin signaling.

**Conclusion:**

Intact progesterone signaling in non-progressive endometrial cancer seems to be an important factor stimulating immunosurveilance and inhibiting transition from an epithelial to a more mesenchymal, more invasive phenotype.

## Introduction

Each year, worldwide, more than 287,000 women develop endometrial cancer making it the most common gynecological cancer in the world and the fourth most common female malignancy in developed countries [1]. Usually endometrial cancer is detected in an early stage and surgery is the cornerstone of treatment. Where there is recurrent or metastatic disease, however, the situation is different. (Neo-)Adjuvant radiation and/or systemic therapy in combination with surgery is usually indicated and in general, progressive disease has a poor prognosis accounting for 74,000 deaths worldwide each year [2], [3]. Prognostic factors for recurrent and metastatic endometrial cancer include surgical FIGO stage, grade of differentiation, histopathological subtype and myometrial and lymphovascular invasion [2], [4], [5], [6], [7].

In several types of cancer, the presence of tumor infiltrating lymphocytes (TILs) has been correlated with improved prognosis, and much research has been performed on this topic [8], [9], [10], [11], [12], [13], [14], [15]. The rationale is that well differentiated cancer evokes an inflammatory response similar to an acute injury which, after sequential infiltration of different dendritic cell populations, eventually results in T-lymphocyte infiltration [16]. Infiltration of TILs as a positive prognostic factor was first described in cutaneous melanoma, where the presence of TILs was predictive for improved survival [8]. Galon et al. in 2006, showed that infiltration of lymphocytes of the adaptive immune system into the center and invasive margin of colorectal cancer was positively correlated with reduced recurrence and improved survival [10]. In 2009 Kilic et al., showed that high levels of TILs within non-small-cell lung cancer correlated with reduced recurrence and enhanced survival [12]. In ovarian cancer, the presence of intratumoral T-lymphocytes was also positively correlated with improved survival and delayed recurrence of the disease [15]. Furthermore, TILs in ovarian cancer were also associated with increased levels of INF-γ, IL2 and chemokines which indicates T-cell activation and attraction [15].

The presence of TILs has not been extensively investigated in endometrial cancer. In endometrial cancer, infiltration of cytotoxic (CD8+) T-lymphocytes in the area of the lesion has been described as an independent prognostic factor and is positively correlated to disease free- and overall survival [17], [18]. In addition, a high cytotoxic T-lymphocyte/regulatory T-lymphocyte (CD8/FOXP3) ratio has been described to be correlated to improved survival in type I endometrial cancer [17].

Next to the influx of T-lymphocytes into the tumor area, the presence of progesterone receptors (PR) is also described as an important asset in prognosis and treatment of endometrial cancer [19], [20], [21]. In well differentiated endometrial cancer PR expression is usually maintained and treatment with medroxyprogesterone acetate (MPA), of those patients with well differentiated disease who chose to preserve fertility, is usually successful [22], [23]. Loss of PR, however, is a negative prognostic factor and is associated with progressive disease in which MPA treatment is usually only temporally successful in 15–20% of cases [24].

Recently, our group has studied the mechanism through which progesterone can induce differentiation during the normal menstrual cycle and can inhibit well differentiated endometrial cancer growth. It was observed that progesterone treatment results in induction of expression of two important inhibitors of Wnt/β-catenin signaling: DKK1 and FOXO1 [25], [26]. In endometrial cancer, activation of Wnt/β-catenin signaling is observed in 30–40% of well differentiated endometrioid carcinomas [27] and progesterone induced inhibition of the Wnt signaling pathway is hypothesized to be an important mechanism to reduce cancer progression [25].

In this study we aimed to investigate the role of progesterone as a direct inhibitor of the migratory capacities of endometrial cancer cells and its role in T-lymphocyte associated inhibition of progressive disease.

## Materials and Methods

### Patient materials

Primary endometrial carcinoma tissue from women with (n = 9) and without (n = 9) a known episode of recurrence or metastasis, was obtained from patients treated between 1997 and 2006 in the University Hospital Gasthuisberg, Catholic University Leuven, Belgium. From this point on, non-recurrent disease is referred as non-progressive disease and recurrent/metastatic disease as progressive disease. Histopathological grading, staging and typing were determined according to the guidelines of the WHO and FIGO [28], [29] and all tumors were revised by a pathologist experienced in gynaecopathology (PCE). Patients with an endometrioid type and a FIGO stage I endometrial carcinoma were included. Patients treated with radio- or chemotherapy prior to surgery, using hormonal steroids or with a second malignancy were excluded. Complete clinical history was obtained from all patients and follow-up was revised to date. Specimens were snap-frozen in liquid nitrogen for RNA-isolation or fixed in formalin and embedded in paraffin for immunohistochemistry (IHC). For microarray analysis, from 4 non-progressive and 4 progressive patients, snap frozen tumor specimens were used. These were chosen because they contained >80% tumor tissue and good quality RNA could be isolated from them. For RT-PCR, 6 non-progressive and 6 progressive snap frozen patient tissue samples were used. For IHC 9 non-progressive and 9 progressive paraffin embedded patient tissue samples were available. Tissue and clinical data collection for the current research study was approved by the Medical Ethical Committee of the University Hospital Gasthuisberg and patients gave written informed consent for tissue collection and clinical data collection for all research purposes.

### Cell culture

For all cell line experiments, Ishikawa endometrial cancer cell lines stably transfected with PRA (IKPRA-1), PRB (IKPRB-1) or PRA and PRB (IKPRAB-36) (previously described by Smit-Koopman et al. [30]) were cultured and maintained in regular culture medium (DMEM/F12 Glutamax, Invitrogen, Carlsbad, CA, USA) in the presence of 5% Fetal Calf Serum (Invitrogen) supplemented with penicillin and streptomycin (Invitrogen). Neomycin (ICN Biomedicals, Costa Mesa, CA, USA) and hygromycin (Invitrogen) 1∶200 were used to maintain selection. For all assays, cells were cultured in DMEM/F12 Glutamax culture medium supplemented with penicillin and streptomycin (Invitrogen), containing 5% charcoal stripped FCS (Invitrogen) with addition of hygromycin and neomycin.

### Immunohistochemistry

IHC studies for CD4 (Sanbio BV, Uden, The Netherlands), CD8 (Dako, Glostrup, Denmark), FOXP3 (Natutech, Frankfurt am Main, Germany) and PRA+PRB (Progesterone Receptor Ab-8, Neomarkers, Fremont, CA, USA) were performed on 4 µm paraffin sections on Starfrost-slides (Knittel, Braunschweig, Germany). Prior to incubation with the primary antibody, the slides were deparaffinized in xylene and rehydrated to 70% ethanol. For CD4+ and CD8+ T-lymphocyte staining, slides were microwaved at 850 Watt in Tris/EDTA pH 9.0 for 15 min. Endogenous peroxidase activity was blocked with 30% H_2_O_2_ in PBS for 5 min. Primary antibodies were applied at respectively 1∶160 (CD4) and 1∶200 (CD8) in Tris/HCl pH 8.0 and incubated at room temperature for 30 min. After washing with Tris/HCl pH 8.0, sections were incubated for 30 min. at room temperature with biotinylated secondary antibody (Dako, 1∶400). After washing with Tris/HCL, the substrate Diaminobenzidine (Dako) was used for visualization of antigen–antibody reactivity.

For FOXP3, slides were blocked (peroxidase deactivation) for 20 min at room temperature (RT) in 30% H_2_O_2_ in methanol and boiled (antigen retrieval) in a citrate-buffer pH 6.0 for 15 min. Primary antibody was applied at 1∶25 and incubated at 4°C overnight. After washing with PBS, slides were incubated for 30 min. with a secondary rabbit-anti-rat antibody (DAKO, 1∶150) and incubated for 30 min. with AB-complex (Dako). The substrate Diaminobenzidine (Dako) was used for visualization of antigen–antibody reactivity.

For PRA+PRB staining, endogenous peroxidase activity was blocked for 5 min at RT in a 10% H_2_O_2_ in methanol solution and the slides were microwaved (antigen retrieval) in a microwave-oven at 850 Watt in 10 nM citric acid buffer pH 6.0 (DAKO) for 15 min. After cooling to room temperature slides were washed with PBS and blocked for 30 min with 0.3% BSA/PBS. Primary antibody was applied at 1∶50 and incubated at 4°C overnight. After washing with PBS, slides were incubated for 30 minutes with a biotinylated secondary goat-anti-mouse antibody (Dako, 1∶400). After the second wash the slides were incubated for 30 min with AB-complex (Dako, 1∶1∶50). The substrate Diaminobenzidine (Dako) was used for visualization of reactivity. All slides were counterstained with hematoxylin for 30 s, then dehydrated and mounted.

For Vimentin staining, a wound-healing assay was performed in 2-well chamber slides (Lab-Tek, Thermo Fisher Scientific, Waltham, MA, USA), in the presence and absence of 1 nM medroxy-progesterone acetate (MPA), and terminated after 48 hr. The cells were washed three times with PBS, fixed with 4% formaldehyde/PBS for 15 minutes and permeabilized with 0,3% Triton100/PBS for 5 minutes. After washing, endogenous peroxidase activity was blocked with 10% H_2_O_2_ in methanol for 5 minutes. Slides were washed and then blocked for 30 minutes with 0.3% BSA/PBS. The anti-vimentin antibody (Invitrogen) was applied at 1∶50 and the slides were incubated for 30 minutes at room temperature. After washing with PBS, slides were incubated with a GFP-fluorescent goat-anti-mouse secondary antibody (Invitrogen) at 1∶500. After washing, the slides were incubated for 5 minutes with DAPI Nucleic Acid Staining Solution (Invitrogen) for nuclear staining. After termination of the reaction with dH_2_O, the slides were mounted and fluorescent images were taken with the Axioplan 2 Imaging Fluorescent Microscope (Carl Zeiss AG, Jena, Germany).

### Counting TILs

After staining, the slides were scanned with the NDP slide scanner (Hamamatsu, Hamamatsu City, Japan) and CD4, CD8 and FOXP3 positive tumor infiltrating lymphocytes (TILs) were counted using Image J software (National Institutes of Health, Bethesda, MD, USA). The number of TILs was determined inside the tumor (Intratumoral), at the tumor edge (Tumor Edge) and at the endometrial/myometrial border (EM). The complete tumor edge and endometrial/myometrial border were evaluated for the presence of TILs. The intratumoral count was performed by counting the TILs in 10 different randomly picked areas (1170 µm×932 µm) chosen by an independent investigator, thereby eradicating observer bias.

### WST1 assay

For the WST1 proliferation assay, IKPRA-1, IKPRB-1 and IKPRAB-36 cell lines were cultured in the absence or presence of MPA in a 96 well plate (Corning Costar, Cambridge, MA, USA). At time 0, the cells were incubated with cell proliferation reagent WST1 (Roche, Basel, Switzerland) for 3 hours at 37°C and absorbance was measured with the Microplate Reader (BIORAD, model 550, Hercules, CA, USA). After baseline measurement the cell lines were cultured in the presence and absence of 1 nM MPA for 96 hours and at 96 hours, the WST1 assay was repeated.

### Migration assays

For the wound-healing assay, IKPRA-1, IKPRB-1 and IKPRAB-36 cell lines were cultured in a 6-well plate (Corning Costar). After inducing the wound, cells were incubated with 1 nM MPA for 96 hours. Wound healing was verified every 24 hr by photography, and analyzed by measuring closure of the wound.

For the modified Boydon assay, cells were seeded in the upper well of a modified Boydon chamber (Transwell, 8 µm pores, 24 mm inserts, 6 well plate, Corning Costar) at 2.5×10^5^ cells per well in the presence or absence of 1 nM MPA. Furthermore as a control, cells were cultured in a Boyden chamber in the presence or absence of 1 nM MPA in combination with 100 nM of the anti-progestagin Org31489 (Organon, Oss, The Netherlands). After 96 hours, cells that had migrated through the filter into the lower well or to the bottom of the insert were trypsinized and counted under the microscope.

### Western blotting

IKPRA-1, IKPRB-1, IKPRAB-36 and IKLV-8 cell lines were cultured in the absence or presence of 1 nM MPA for 96 hrs and subsequently lysed at 0°C in Cell Lysis Buffer (Cell Signaling Technology, Danvers, MA, USA) for 5 minutes. Then the cells were scraped, centrifuged at 14.000 rpm for 10 minutes and the supernatant was removed. The protein concentration was calculated using the Protein Assay Kit (Pierce, Thermo Scientific, Rockford, IL, USA) and of each sample 4.5 µg protein in 30 µL lysisbuffer+BSA was loaded on a 10% SDS-PAGE gel. Western blotting was performed according to standard procedures. The blotting paper was blocked for 30 minutes at RT with Blocking Buffer (LI-COR Biotechnology, Lincoln, NE, USA) and then incubated overnight at 4°C using rabbit polyclonal anti-hFOXO1 antibody (1∶5000, Bethyl Laboratories, Montgomery, TX, USA) in Blocking Buffer (LI-COR Biotechnology). Next, the blotting membrane was incubated with the secondary goat-anti-rabbit IgG (IRDye 800CW, 1∶5000, LI-COR Biotechnology) for 30 minutes at RT and washed. As a loading control, the membrane was incubated for 30 minutes with the monoclonal anti-β-actin (1∶1000, Sigma-Aldrich, Saint Louis, MO, USA), washed with PBS and incubated for 30 minutes with the secondary goat-anti-mouse IgG (IRDye 680CW, 1∶5000, LI-COR Biotechnology). The specific protein bands were detected using the Odyssey Scanning System (LI-COR Biotechnology).

### RNA-isolation, gene expression analyses and quantitative real-time RT-PCR

Patient tissue samples were sectioned (5 µm, cryostat) and every 10^th^ section was HE stained and revised by the pathologist (PCE) to assess tumor load. Only sections containing >80% tumor were lysed in Trizol (Invitrogen) and sonified for 1 min. The PRA and PRB expressing Ishikawa cell line (IKPRAB-36) was cultured for 48 h in the absence or presence of 1 nM MPA (n = 3), placed on ice and lysed in Trizol (Invitrogen).

Phase separation was accomplished with 0.2 ml chloroform and centrifugation for 15 min. The supernatant was transferred and isopropanol was added for RNA precipitation. The precipitated RNA was washed with 75% ethanol. All RNA was cleaned with the Rneasy Minelute cleanup kit (Qiagen, Venlo, The Netherlands). Amount and quality of the RNA was assessed by using the Nanodrop (Nanodrop, Wilmington, DE, USA) and Bio-analyzer (Aligent, Santa Clara, CA, USA).

RNA isolated from patient and cell line material was labeled according to Affymetrix labeling protocols and labeled RNA was applied to genome-wide expression arrays (Affymetrix U133plus2 GeneChips containing 54,614 probe sets, representing approximately 47.000 transcripts (Affymetrix, Santa Clara, CA, USA)). Using RMA (Robust Multi-array Analysis [31]), normalization of raw data was performed to be able to produce gene lists and eventually calculate significantly regulated genes using SAM (Stanford University, Stanford, CA, USA [32]). Lists of SAM regulated genes (1.25 fold or more; delta-values resembling p<0.05) were loaded in the Ingenuity pathway assist software to assess the involvement of different biological pathways (Ingenuity, Redwood City, CA, USA). For the patient materials raw lists of regulated genes (1.25 fold or more) were loaded in Ingenuity.

All micro-array data is MIAME compliant and raw data has been deposited in the MIAME compliant GEO database under series: GSE29437 (consisting of GSE29435: cell line data; and GSE29436: patient data).

Genes for quantitative real-time RT-PCR were identified by micro-array analysis and pathway analysis. RNA was transcribed into cDNA with the use of the Affymetrix one-cycle cDNA synthesis kit (Affymetrix). For identified genes, primers were ordered and tested (a list of primers is included in Table S1). The housekeeping gene β-actin was used as a reference gene. RT-PCR was performed and analyzed using the CFX RT-PCR system (Bio-Rad, Veenendaal, The Netherlands).

### Statistics

For the statistical analyses of the CD4+, CD8+ and FOXP3+ cell counts, modified Boyden chamber assay data, WST1 assay data and RT-PCR data, SPSS 15.0 was used (IBM, Armonk, NY, USA). For normal distributed data a t-test and for skewed data a Mann-Whitney U-test was performed to assess P-values. A P-value<0.05 was considered statistically significant. To calculate the p-value of regulated pathways, Ingenuity pathway assist software uses a Fisher's exact test.

## Results

### Patient characteristics (Table 1)

**Table 1:** Clinical characteristics of the included patients.

			*Non-progressive (n = 9)*	*Progressive (n = 9)*	*P-value*
			*Patients 1–9*	*Patients 10–18*	
Age - year					p = 0,606
		Mean	68,5	68,6	
		Range	54–85	59–73	
BMI					p = 0,284
		Mean	28,3	32	
		Sd	6,1	4,7	
Histological type	no. (%)				
		Endometrioid	9 (100)	8 (88,9)	
		Mixed	0 (–)	1 (11,1)	
FIGO stage	no. (%)				
		Ia	4 (44,4)	7 (77,8)	
		Ib	5 (55,6)	2 (22,2)	
Tumor grade	no. (%)				
		1	2 (22,2)	5 (55,6)	
		2	3 (33,3)	1 (11,1)	
		3	4 (44,5)	3 (33,3)	
Current status	no. (%)				
		NED	8 (88,9)	3 (33,3)	
		DOD	1 (11,1)	6 (66,7)	
Recurrence	no. (%)				
		No	9 (100)	0 (–)	
		Yes	0 (–)	9 (100)	
Metastasis	no. (%)				
		No	9 (100)	5 (55,6)	
		Yes	0 (–)	4 (44,4)	
Chemotherapy	no.		0	0	
Radiotherapy	no.		0	1	

Table 1 shows the characteristics of the patients included in the study. A p-value of <0.05 was considered as statistically significant. BMI = body mass index; NED = no evidence of disease; DOD = death of disease.

Patients with (n = 9) and without (n = 9) progressive endometrial cancer were included. All included patients underwent primary total abdominal- or laparoscopically assisted vaginal hysterectomy and a bilateral salpingo-oophorectomy combined with lymph node removal. None of the women received chemotherapy and only one woman in the progressive disease group was given radiotherapy after surgery. Histopathological subtypes were endometrioid (n = 17) and mixed endometrioid/mucinoid (n = 1). Tumor grades were 1 (n = 7), 2 (n = 4) and 3 (n = 7) and FIGO stages were Ia (n = 11) and Ib (n = 7). In the progressive disease group all 9 patients had one or more episodes of local recurrence and 4 patients developed one or multiple distant metastases. Recurrences were vaginal, pelvic or (retro)peritoneal, and metastatic sites were the lungs (n = 3), liver (n = 1), spleen (n = 1) and brain (n = 1). Clinical follow-up to date was available for all patients. In the non-progressive group 8 patients are currently free of disease and 1 patient died in follow-up. In the progressive disease group 3 patients are free of disease and 6 patients died from their endometrial cancer related disease. Patient characteristics are detailed in Table 1.

### Progesterone receptor status and detection of CD4+ T-helper, CD8+ cytotoxic T-cells and FOXP3+ regulatory T-cells in non- progressive and progressive disease

The presence of tumor infiltrating lymphocytes has been correlated to prolonged survival in endometrial cancer [17], [18]. Furthermore, loss of progesterone receptor (PR) expression in endometrial cancer has been found to be a risk factor for progressive disease [33]. In order to substantiate the relationship between intact PR signaling and the presence of infiltrating lymphocytes in non-progressive disease, immunohistochemical staining and, when appropriate, quantitative measurements were performed.

**Figure 1:**
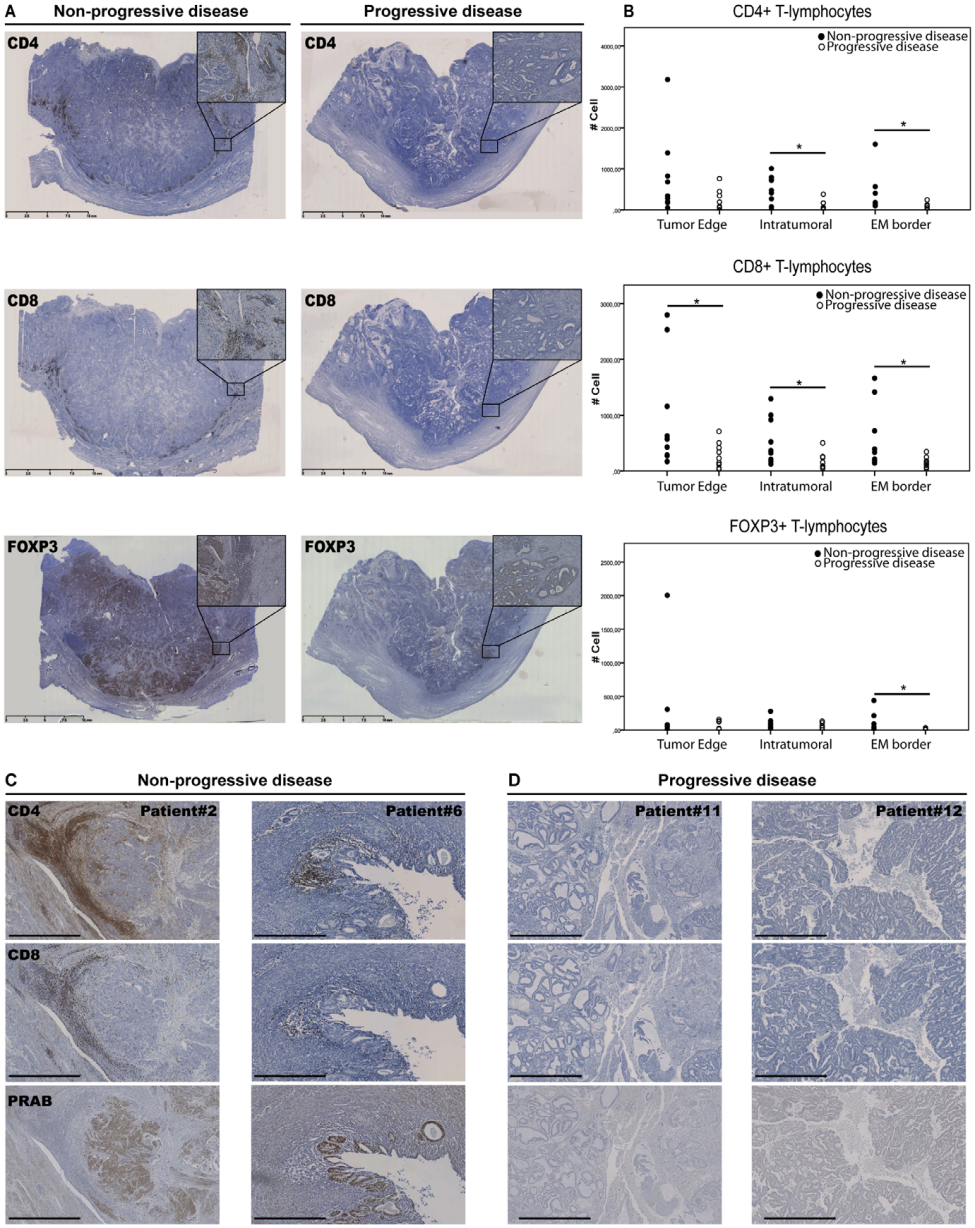
Expression and histological distribution of PRA+PRB and CD4+, CD8+ and Foxp3+ T-lymphocytes in primary endometrial carcinoma specimens. A: Overview of immunohistochemical staining for CD4, CD8 and FOXP3 in primary endometrial cancer specimens in non-progressive disease (n = 9) compared to progressive disease (n = 9) (magnification 0,4x, inlay 10x). Non-progressive disease shows pronounced staining, whereas progressive disease shows reduced staining. The scale-bar represents 10 mm. B: Quantification of CD4, CD8 and FOXP3 cell counts on the tumor edge (Tumor Edge), in the tumor (Intratumoral) and on the endometrial-myometrial border (EM border). *indicates a p-value<0.05 (Mann-Whitney U-test). C and D: Representative non-progressive (C) and progressive (D) patient tissues were stained for CD4, CD8 and PRA+PRB and show a positive correlation between the presence of TILs and the expression of PR. Magnification is 5x and the scale-bar represents 1 mm. Patients 6 and 11 were both included in the micro-array analyses. Furthermore patient 11 had only recurrent disease, while patient 12 had recurrent and metastatic disease.

As exemplified in Fig. 1A, in progressive disease immunohistochemical staining for CD4+, CD8+ and FOXP3+ T-lymphocytes seems reduced as compared to staining in non-progressive disease. Quantification of the number of CD4+, CD8+ and FOXP3+ T-lymphocytes in progressive disease indeed confirmed a lower number of positive cells located on the endometrial-myometrial border (Fig. 1B, EM), at the edge of the tumor (Fig. 1B, Tumor Edge) and within the tumor (Fig. 1B, Intratumoral). Whether the reduced cell counts were significantly different between the non-progressive and progressive endometrial cancer tissues is indicated in the Figure (Fig. 1B).

Furthermore, reviewing consecutive sections in non-progressive disease for expression of progesterone receptors (PR) revealed that the presence of CD4+ and CD8+ T-lymphocytes was positively correlated with the presence of PR staining (Fig. 1C and 1D).

### Genome-wide expression analyses of primary endometrial carcinoma tissue

To investigate whether the correlation between PR signaling and the presence of tumor infiltrating lymphocytes could indicate a causative relationship, a genome-wide mRNA expression analysis on snap-frozen primary endometrial carcinoma specimens from 4 patients without and 4 patients with progressive disease was performed. At the individual gene level it was observed that a marked number of chemokines and cytokines were differentially regulated between non-progressive and progressive disease (Table S2). For example, the chemokines CCL21 (−1.5x), CXCL9 (−2.9x), CXCL10 (−2.1x) and CXCL14 (three data sets present: −33.0x; −20.5x; −6.4x, respectively) were all down regulated in progressive disease while the cytokines IL8 (2.0x; 5.7x; 9.5x) and IL32 (1.9x) were up-regulated in progressive disease (Table S2). Furthermore, earlier work from our group has indicated activation of Wnt/β catenin signaling in progressive disease [25] and in agreement with this a number of Wnt/β-catenin inhibitory- and target genes were lost from progressive disease (DKK1, DKK4 and WIF1) (Table S2).

Interestingly, a number of the above mentioned genes which were down-regulated in progressive disease, have been described in literature to be up-regulated by progesterone (CXCL14 [34], DKK1 [25], MMP7 [35] and SFRP4 [36]). This is in agreement with the finding that PR expression (at protein and mRNA expression level (Fig. 1C and 1D and Table S2) is down regulated in progressive disease.

Upon reviewing pathways regulated between non-progressive and progressive disease, regulation of a number of pathways involved in carcinogenesis and invasive disease and involved in immunosurveillance was found to be significantly regulated: Integrin Signaling, Molecular Mechanisms of Cancer, Antigen Presentation Pathway, Non-Small Cell Lung Cancer Signaling, IGF-1 Signaling, Role of Tissue Factor in Cancer, Leukocyte Extravasation Signaling, ERK/MAPK Signaling, Colorectal Cancer Metastasis Signaling (which includes Wnt/β catenin signaling), FGF Signaling, FAK Signaling, etc (the complete list of regulated pathways and their consecutive p-values can be accessed from Table S3).

**Figure 2:**
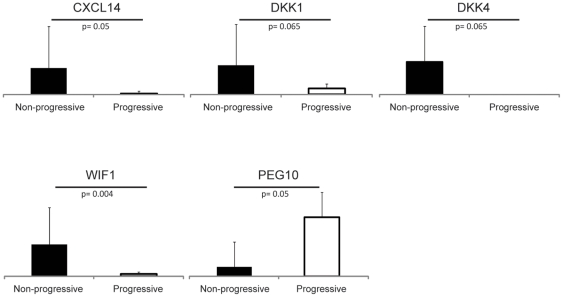
RT-PCR results of genes of interest in the patient samples. CXCL14, DKK1, DKK4, WIF1 and PEG10 were selected from the micro-array results and verified with real time RT-PCR. Significance was calculated using a Mann-Whitney U-test. A p-value of 0.05 was considered to be statistically significant.

For a number of genes (CXCL14, DKK1, DKK4, PEG10 and WIF1) a quantitative real-time RT-PCR was performed in order to verify regulation (Fig. 2).

### Effect of progesterone on migration of the Ishikawa endometrial cancer cell lines

In order to further corroborate the possible role for progesterone in regulating invasion, Ishikawa endometrial carcinoma cell lines stably transfected with PRA, PRB, or PRA and PRB [30] were cultured in the presence or absence of MPA for varying periods of time and used in two different experiments measuring cell migration. To verify cell proliferation during the different experiments a WST1 proliferation test was performed which showed that within the indicated timeframe no significant differences in proliferation could be detected between cells incubated with or without MPA.

**Figure 3:**
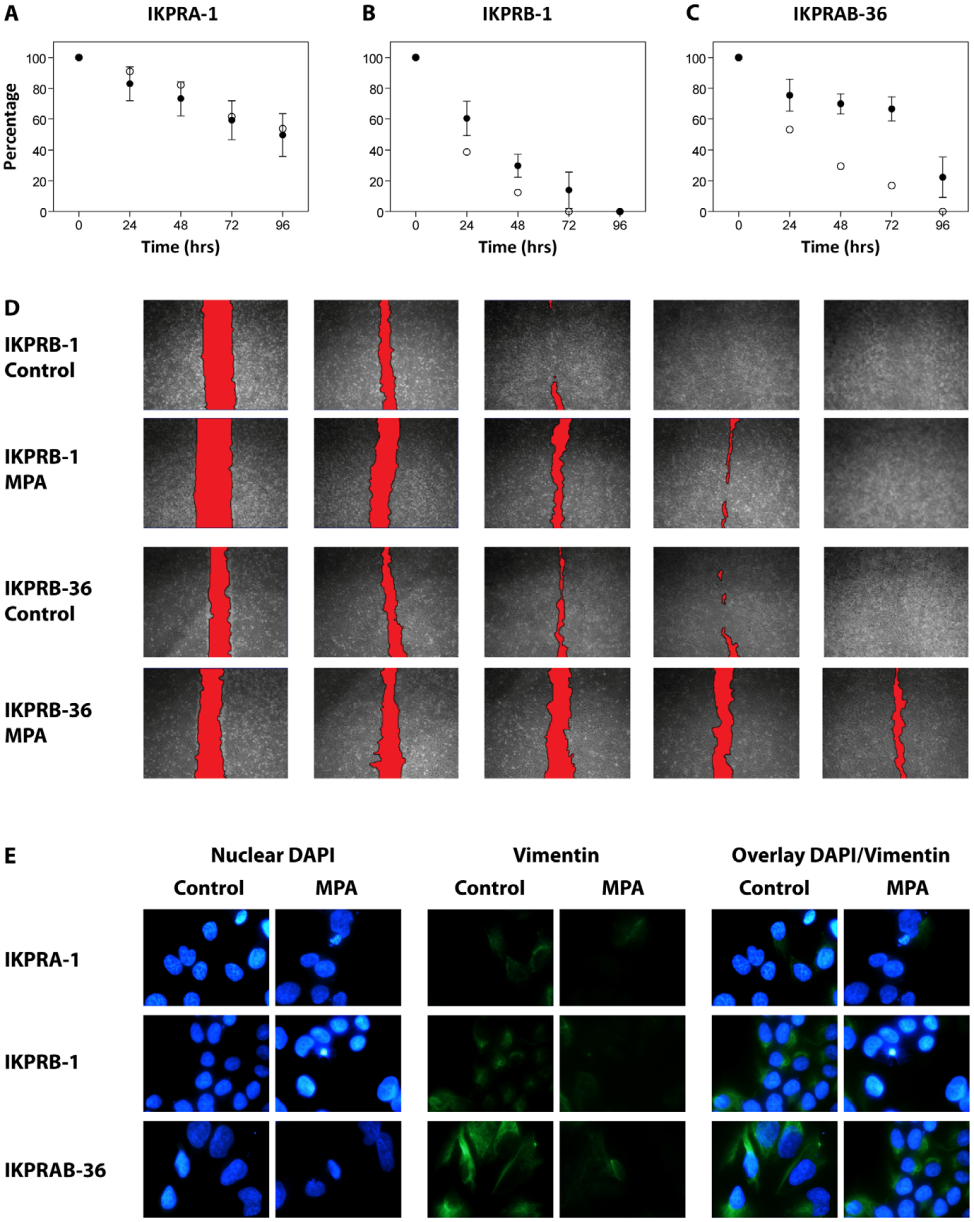
Progesterone induced inhibition of migration in a wound-healing assay. IKPRA-1 (A), IKPRB-1 (B) and IKPRAB-36 (C) cells were cultured in the absence (white bullets) or presence (black bullets) of 1 nM MPA and used for a wound-healing assay (n = 3) and closure of the wound was measured as a percentage of total closure (100% means the wound is open, 0% means the wound has closed). D shows representative images of the process of wound-healing with in red the wound. E shows IF for nuclei (DAPI) and vimentin expression on the invasive front of the manually inflicted wound. In this figure, the wound was always situated on the right side.

In Figure 3, different Ishikawa cell lines were subjected to a wound-healing assay in the presence or absence of MPA (1 nM) for up to 96 h. It was observed that, in the stably PRB expressing (IKPRB-1) and PRA+PRB expressing (IKPRAB-36) Ishikawa cell lines, MPA inhibited closure of the manually inflicted wound (Fig. 3A–D). Furthermore, when we stained the edge of the wound for the mesenchymal marker vimentin, it was observed that in the presence of MPA vimentin expression was clearly reduced (Fig. 3E). Next to this detail on expression of vimentin, the overall vimentin levels were decreased in IKPRB-1 and IKPRAB-36 cell lines incubated with 1 nM MPA. It was also observed that in the stably PRA expressing (IKPRA-1) Ishikawa cell line, neither wound healing nor vimentin expression was affected by MPA (Fig. 3A and 3E).

**Figure 4:**
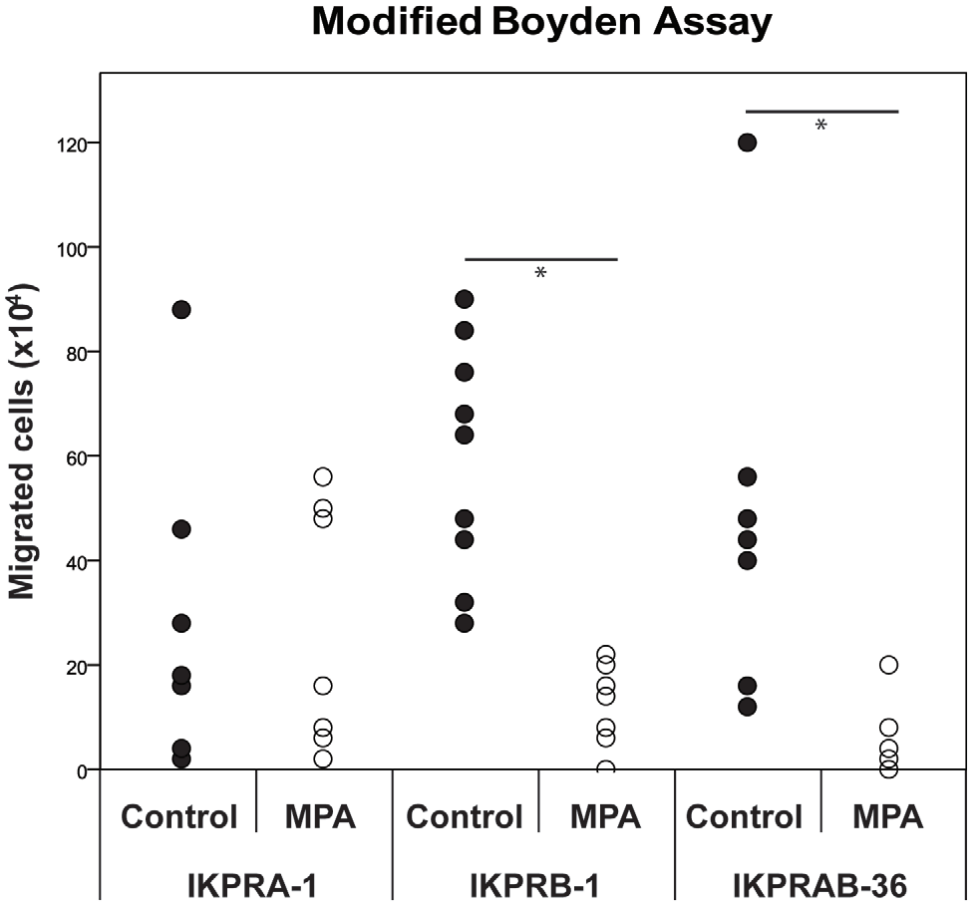
Invasion of PR positive Ishikawa EC cell lines. IKPRA-1, IKPRB-1 and IKPRAB-36 cells were cultured in the absence (black dots) or presence (white dots) of 1 nM MPA in a modified Boyden chamber. After 96 hours, cells that had migrated through the pores of the upper well were counted. The figure represents three independent experiments performed in triplicate. *indicates a p-value of <0.05 (Mann-Whitney U-test).

In Figure 4, another approach was used to study the migratory capacity of different Ishikawa cell lines in the presence or absence of progesterone. It was observed that for IKPRB-1 as well as IKPRAB-36 cells, migration in a modified Boyden chamber was inhibited in the presence of progesterone. Furthermore, for the IKPRA-1 cell line such a differential regulation of migration under the influence of MPA was not observed.

### Genome-wide expression analysis of Ishikawa endometrial cancer cell line

To further document progesterone-induced inhibition of cellular migration and to investigate the involvement of progesterone signaling in T-lymphocyte infiltration, IKPRAB-36 cells were cultured for 48 h in the presence or absence of 1 nM MPA and used for genome-wide expression analysis. It was observed that 1616 genes were significantly regulated by progesterone in the IKPRAB-36 cell line (1029 up-regulated, 587 down-regulated, Table S4).

**Figure 5:**
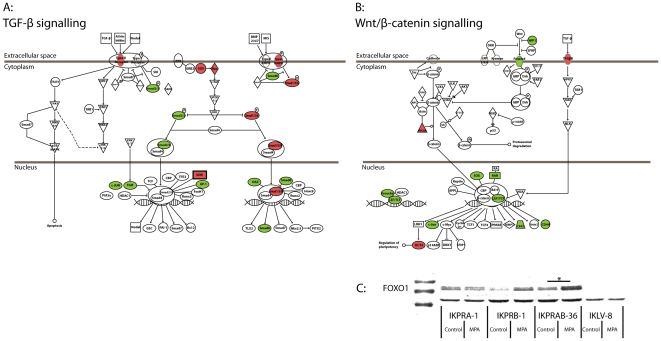
MPA induced regulation of TGF-β and Wnt/β-catenin signaling in the IKPRAB-36 cell line. A and B: In these pathways a green color represents down regulation by MPA and a red color represents up regulation by MPA. Signaling pathways were provided by Ingenuity Pathway Assist Software© and individual gene expression levels are available in Table S4. C: Western blot showing FOXO1 expression in the IKPRA-1, IKPRB-1, IKPRAB-36 and IKLV-8 cell lines cultured in the absence (control) or presence (MPA) of 1 nM MPA. *indicates significant regulation in the micro-array analysis (Table S4).

Using Ingenuity pathway analysis of significantly regulated genes, the following pathways were observed to be regulated by progesterone (the complete list of regulated pathways and their consecutive p-values can be accessed from Table S5): IGF-1 signaling, Neuregulin signaling, TNFR1 signaling, P13K signaling in B-lymphocytes, VDR/RXR signaling, Acute Phase Response signaling, Hepatic Fibrosis/Hepatic Stellate Cell activation, Molecular Mechanisms of Cancer (which includes Wnt/β-catenin and TGF-β signaling), TGF-β signaling, Axonal Guidance Signaling etc. Interestingly, it was noted that 41/67 pathways observed to be significantly regulated by progesterone in the cell line were also found to be significantly regulated between non-progressive and progressive disease (see Table S6). Furthermore, it was also noted that a number of pathways specifically involved in transition from a epithelial state to a mesenchymal state (EMT) was significantly regulated by progesterone and in the endometrial cancer samples: EGF signaling (p = 0.029), IGF-1 signaling (p = 0.0000006), IL-6 signaling (0.013), ILK signaling (p = 0.018), PDGF signaling (p = 0.03), TGF-β (p = 0.003), VEGF signaling (p = 0.022) and Wnt/β-catenin signaling (p = 0.036). In Figure 5A and B, MPA-induced gene regulation in Wnt/β-catenin and TGF-β signaling is shown. Next to this, a heat map confirmed a major overlap between gene regulation by MPA and differential gene expression between non-progressive and progressive disease (Table S7).

Regulation of the Wnt signaling pathway was further confirmed by showing progesterone induction of the Wnt inhibitor FOXO1 at the protein level (Fig. 5C).

## Discussion

In general, patients with endometrial cancer have a good prognosis since early diagnosis is frequent and the disease has usually not spread beyond the uterus. However, the prognosis for recurrent or metastatic endometrial cancer remains poor and in order to improve therapy it is vital to understand the processes which inhibit and stimulate cancer progression.

Infiltration of T-lymphocytes into the region of the lesion, for example, is an anticancer signal which helps to confine a tumor until cancer-induced T-cell death establishes tumor immune tolerance opening the road to progression. The transition of an epithelial phenotype towards a more mesenchymal phenotype is a subsequent step which leads to further progression to invasive disease. Central to this epithelial to mesenchymal transition (EMT) is the activation of important signaling pathways such as Wnt/β-catenin and TGF-β [37]. Activation of these pathways results in induction of Snail1/2 induced transcription, eventually causing degradation of the basement membrane by induction of matrix metalloproteinases, loss of epithelial markers such as E-cadherin and gain of mesenchymal markers such as vimentin [37].

In the current investigations non-progressive and progressive primary endometrial cancer tissues were compared and it was observed that progression of disease was characterized by 1. Loss of progesterone signaling, 2. Loss of CD4, CD8 and FOXP3 T-lymphocytes driven immunosuppression and 3. Modulation of genes and pathways reminiscent of EMT. The aim of the present investigations was to assess the role of decreased progesterone signaling in progressive disease, and more particularly in relation to loss of immunosuppression and transition from an epithelial phenotype to a more invasive mesenchymal phenotype.

### Loss of PR expression correlates with loss of immunosupression and increased EMT in progressive disease

Measuring tumor infiltrating lymphocytes (TILs) in primary endometrial cancer tissues from non-progressive and progressive disease indicated that in patients with non-progressive endometrial cancer, TILs were abundantly present. This is in agreement with studies by Kondratiev et al. in 2004 [18] and De Jong et al. in 2009 [17], which showed that high levels of CD8+ T-lymphocytes were associated with improved disease free survival. Furthermore, the presence of several chemokines (CCL21, CXCL9, CXCL10, CXCL14, IL8 and IL32) indicated that there is an active process which directs TILs to the site of the lesion [38]. Interestingly, a number of these chemokines are up-regulated during the secretory phase of the menstrual cycle when progesterone levels are increased (CCL21: 1.5-fold up, CXCL10: 1.3-fold up and CXCL14: 90-fold up; [39]). Furthermore, CXCL14 has also been described by other groups to be a progesterone induced gene in the endometrium involved in chemo-attraction of uterine natural killer cells to the epithelial glands [34]. In summary, this indicates a putative role for progesterone signaling in attracting TILs in non-progressive endometrial cancer.

In the patient tissues which were used in the current investigations, progesterone receptor expression was lost from progressive disease. The fact that hormonal control of a tissue is lost upon progressive malignant transformation is not a new finding and besides loss of PR expression in endometrial cancer [20] this has also been described for other cancer types like breast cancer (loss of estrogen signaling [40]) and prostate cancer (loss of androgen signaling [41]) as well.

According to previous work from our group, besides stimulating TILs, progesterone can inhibit Wnt/β-catenin signaling and loss of progesterone signaling may be involved in tumor onset and progression towards a more invasive disease [21], [25], [42], [43]. Interestingly, upon reviewing gene expression profiles obtained from progressive and non-progressive endometrial cancer, a number of inhibitors of Wnt/β-catenin signaling were indeed found to be down-regulated in progressive disease (DKK1, DKK4 and WIF1). These findings are in accordance with the hypothesis that Wnt/β-catenin signaling becomes activated through loss of PR signaling, thus accommodating progressive disease [25]. Down-regulation of the Wnt/β-catenin signaling inhibitor WIF1, in this respect, is of interest because down regulation of WIF-1 in prostate cancer cells was observed to be associated with an increased capacity for cell migration and invasion [44]. In keeping with this, in colorectal cancer, overexpression of activated nuclear β-catenin (the hallmark of activated Wnt/β-catenin signaling) is located at the invasive front of the tumor [45] and in colorectal cancer cell lines, activation of β-catenin directly induces EMT [46].

PEG10 was found to be significantly up regulated in progressive disease. Interestingly, PEG10 is a biomarker for progressive development and invasion of hepatocellular carcinoma, gallbladder adenocarcinoma and acute lymphoid leukemia and is found to be regulated by androgens [47], [48], [49], [50]. Next to this, PEG10 and IL10 expression is activated by ligation of CCL10-CCR7 and CXCL13-CXCR5 in B-cell acute lymphatic leukemia, and PEG10 contributes to the up-regulation of IL10, which can lead to impairment of the cytotoxicity of CD8+ T-lymphocytes [51]. It was observed that CXCL13 (3,17x) and PEG10 (9,38x and 4,38x, p = 0,05) were both up-regulated in progressive disease and possibly this up-regulation can contribute to impairment of the T-lymphocyte mediated anti-tumor response in progressive disease.

Upon reviewing other pathways which were differentially expressed between non-progressive and progressive endometrial cancer, significant up-regulation of a number of pathways involved in progression towards a more mesenchymal phenotype was noted (Table S3). IL8 signaling is one of those regulated pathways and IL8 itself was found to be up regulated 9.5-fold in progressive disease. These data are in line with literature showing that IL8 is a progesterone down-regulated gene [52] and that high levels of IL8 correlate with endometrial metastatic disease [53].

### MPA inhibits EMT in the Ishikawa endometrial cancer cell line

In order to further substantiate the above finding that loss of progesterone signaling in progressive disease may play a role in diminished T-cell infiltration and induction of EMT, progesterone signaling in the well differentiated Ishikawa endometrial cancer cell line was investigated.

Although both PRA and PRB can activate transcription of target genes in response to progesterone, PRA and PRB have different transcriptional activities [54]. It has been documented that PRB is a stronger activator of transcription than PRA and PRA is thought to be a dominant repressor of PRB [55]. Next to this, the difference in transcriptional activity is further explained by the recruitment of different cofactors by PRA and PRB [56], [57].

In the present study, it was observed that culture of the IKPRB-1 and IKPRAB-36 endometrial cancer cell line, but not IKPRA-1, in the presence of MPA resulted in inhibition of migration and down regulation of the mesenchymal marker vimentin at the edge of a manually inflicted wound.

These findings suggest that progesterone, in vitro, can inhibit cancer cell migration due to inhibition of EMT. Assessment of pathways involved in EMT showed progesterone modulated down regulation of EGF, IGF-1, IL-6, Integrin/ILK, PDGF, TGF-β, VEGF and Wnt/β-catenin signaling. Interestingly, all of these pathways were also observed to be modulated in progressive disease (Table S6). As shown, many of the observed altered signaling pathways in the patient samples (Table S3) were also significantly altered in the Ishikawa cell line, when incubated with or without progesterone (Table S5). In the Ishikawa culture obviously no tumor infiltrating lymphocytes are present and it is only progesterone signaling that contributes to these changes in signaling. Therefore we conclude that regulation of signaling pathways in patient samples can not only be attributed to the presence or absence of tumor infiltrating lymphocytes, but also to changes in progesterone receptor signaling.

Progesterone inhibition of TGF-β signaling and induction of TGF-β signaling in progesterone insensitive progressive disease is an interesting finding because enhanced TGF-β signaling has been shown to be a very potent immunosuppressant signal used in transplantation medicine. Several agents inhibiting TGF-β signaling (anti-TGF-beta antibodies, small molecule inhibitors of TGF-beta, Smad inhibitors) are in the early stages of development aiming to alleviate immunosuppression during carcinogenesis [58]. Furthermore, neutralizing TGF-β resulted in a CD8+ T-lymphocyte anti-tumor immune response in mouse models [59].

Enhanced TGF-β signaling is also of interest because it has been described as an important major driving force of EMT. Reviewing the pathway in more detail revealed for example up regulation of cell adhesion molecule L1CAM. For L1CAM, regulation of transcription by TGF-β signaling has been described [60], but, interestingly, in colorectal cancer L1CAM has also been shown to be a target gene of Wnt/β-catenin signaling and expression of L1CAM was found to co-localize with β-catenin in the invasive front of the tumor [61]. Recently, for endometrial cancer similar observations have been described confirming promoter-binding sites for the Wnt/β-catenin inducing transcription factor LEF-1 and, interestingly, also for the EMT inducing transcription factors SNAI1 and SNAI2 [60].

In summary, intact progesterone signaling in non-progressive endometrial cancer seems to be an important factor stimulating immunosuppression and inhibiting transition from an epithelial to a more mesenchymal, more invasive phenotype.

## Supporting Information

Table S1 **Primers of genes of interest used for RT-PCR in endometrial carcinoma samples.** List of used primers for the q-PCR experiments.
(XLS)

Table S2 **List of differentially expressed genes in endometrial carcinoma patient samples.** Differentially regulated genes between non-progressive (n = 4) and progressive (n = 4) endometrial cancer samples. Negative values indicate down regulation in progressive disease, positive values indicate up regulation in progressive disease. A fold chance of +/−1.25 was used as a cutoff point.
(XLS)

Table S3 **List of differentially regulated pathways in progressive versus non-progressive endometrial cancer patients.** A list of differentially regulated genes in the progressive group was entered in Ingenuity pathway analysis software. A fold chance of +/−1.25 was used as a cutoff point. P-values were calculated with a Fishers exact test and a p-value<0.05 was considered statistically significant.
(XLS)

Table S4 **List of significantly MPA regulated genes in the IKPRAB-36 endometrial cancer cell line.** List of significantly MPA regulated genes in the Ishikawa IKPRAB-36 cell line (n = 3). Negative values indicate down regulation by MPA, positive values indicate up regulation by MPA. A fold chance of +/−1.25 was used as a cutoff point and the delta value was 0.53, which resembles p<0.05.
(XLS)

Table S5 **List of MPA regulated pathways in the IKPRAB-36 endometrial cancer cell line.** A list of significantly MPA regulated genes was entered in Ingenuity pathway analysis software. A fold change of +/−1.25 was used as cutoff point. Ingenuity uses a Fishers exact test for calculate significance and a p-value of <0.05 was considered statistically significant.
(XLS)

Table S6 **Pathways significantly regulated in the IKPRAB-36 cell line and in endometrial cancer patient samples.** A p-value of <0.05 was considered to be statistically significant. A grey colored pathway resembles a known EMT associated pathway.
(XLS)

Table S7 **List and heat map of genes both regulated in the IKPRAB-36 cell line and in endometrial cancer patient samples.** List of genes both regulated by MPA in the Ishikawa IKPRAB-36 cell line (n = 3) and differentially regulated between non-progressive versus progressive disease. Negative values indicate down regulation by MPA in IKPRAB-36 cells and in non-progressive as compared to progressive disease, positive values indicate up regulation by MPA in IKPRAB-36 cells and in non-progressive as compared to progressive disease. A fold chance of +/−1.25 was used as a cutoff point.
(XLS)
